# Oligometastatic Mixed Neuroendocrine Adenocarcinoma of the Esophago-Gastric Junction: A Case of Successful Multidisciplinary Management, the Lessons Learnt and Review of the Literature

**DOI:** 10.3390/jcm14051503

**Published:** 2025-02-24

**Authors:** Anastasia Sotiropoulou, Maria Avgoustidou, Vassilis Milionis, Ioannis Papadimitriou, Chrysovalantis Vergadis, Dimitrios Schizas, Nikolaos Arkadopoulos, Orestis Lyros

**Affiliations:** 1 Fourth Department of Surgery, Attikon University Hospital, National and Kapodistrian University of Athens, 12462 Chaidari, Greece; anastasias.234@gmail.com (A.S.); narkado@hotmail.com (N.A.); 2 Department of Oncology, Athens Medical Center, 15123 Marousi, Greece; avgoustidou.oncologist@yahoo.com; 3 First Department of Pathology, National and Kapodistrian University of Athens, 11527 Athens, Greece; milionisv@yahoo.com; 4 Department of Gastroenterology, Athens Medical Center, 15123 Marousi, Greece; ioannispapad@gmail.com; 5 Department of Interventional Radiology, Laikon General Hospital, 11527 Athens, Greece; valvergadis@gmail.com; 6 First Department of Surgery, Laikon General Hospital, National and Kapodistrian University of Athens, 11527 Athens, Greece; schizasad@gmail.com

**Keywords:** mixed neuroendocrine–non-neuroendocrine neoplasm, esophagogastric junction, oligometastatic, multidisciplinary treatment

## Abstract

**Background:** Mixed neuroendocrine–non-neuroendocrine neoplasms (MiNENs) of the esophago-gastric junction (EGJ) are rare aggressive malignant neoplasms, with, currently, limited evidence regarding the appropriate therapeutic approach. **Methods:** Herein, we report multimodal treatment management of a patient with oligometastatic MiNEN of EGJ (Siewert III), discuss the lessons learnt, and provide a review of the literature. **Results:** A 69-year-old female was diagnosed with a locally advanced EGJ tumor and three liver metastases (cT4, cN+, M1). Although the initial histology from biopsy revealed adenocarcinoma, the histopathology of a lymph node biopsy from staging laparoscopy revealed infiltration of neuroendocrine carcinoma cells. Thus, the diagnosis of a mixed neuroendocrine adenocarcinoma was set, and systemic chemotherapy with etoposide and cisplatin was initiated. A major clinical response led to conversion surgical resection of the primary tumor and metastases, followed by adjuvant therapy with immunotherapy. The patient is free of disease at the 3-year follow-up. A review of the literature on similar cases of EGJ or gastric MiNENs revealed a limited number of cases. Out of the 39 patients, 20 of them (51.3%) suffered from advanced-stage disease. The MiNEN diagnosis typically occurred after surgical resection. Systemic chemotherapy against the neuroendocrine component demonstrated significant response rates, while in cases in which conversion surgery was offered, prolongation of survival was demonstrated. **Conclusions:** Our case and the existing literature on MiNENs of EGJ underline the need for a personalized treatment approach following thorough interpretation of comprehensive pretherapeutic staging. Conversion radical surgery with curative intent could be considered in cases of major or complete clinical response to induction chemotherapy with potentially favorable outcomes.

## 1. Introduction

Mixed neuroendocrine–non-neuroendocrine neoplasms (MiNENs) present a group of malignant neoplasms located at the gastro-entero-pancreatic tract, characterized by great heterogeneity regarding their molecular, pathological, and clinical profile [1]. Since the first description of mixed tumors by Cordier in 1924 [2], as tumors consisting of an endocrine and an exocrine compound, several attempts to classify them have been made. In 2016, Rosa et al. proposed the reclassification of MANECs to MiNENs, replacing the term ‘exocrine’ with ‘non-neuroendocrine’ as an umbrella term for histological variants [3]. This term was also used in the fifth edition of the WHO classification of endocrine tumors, released in 2022. To fall into the definition of MiNEN, each component should be at least 30% of the total tumor volume [4,5].

The origin of MiNENs is thought to be a totipotent stem cell present in the tissue submucosa that can differentiate into various cell lines [6]. In most MiNENs, both the neuroendocrine and non-neuroendocrine components are poorly differentiated. Additionally, the mitotic rate and Ki-67 proliferation index of the neuroendocrine component usually fall into the same range as a neuroendocrine carcinoma (NEC). However, in some MiNENs, either (or both) components could be well-differentiated, so they should be graded separately, if possible. These indexes provide valuable information regarding how fast cells proliferate, and have potential diagnostic, prognostic, and predictive roles [7]. MiNENs are treated similarly to NECs, specifically when the neuroendocrine component exceeds 30% of total tumor volume and appears to be the most aggressive and metastatic component. According to the latest edition of the WHO, the classification of neuroendocrine neoplasms of the gastrointestinal (GI) tract takes into consideration the differentiation, grade, mitotic rate (MR) (mitoses/2 mm^2^), and Ki-67 index (%). Accordingly, GI neuroendocrine neoplasms are categorized as follows: neuroendocrine tumor (NET), G1: well differentiated, low grade, MR < 2 mitoses/2 mm^2^, ki67 < 3%, NET, G2: well differentiated, intermediate grade, MR: 2 to 20 mitoses/2 mm^2^, Ki67: 3 to 20%, NET, G3: well differentiated, high grade, MR: >20 mitoses/2 mm^2^, Ki67: >20% (often >70%), NEC, small cell type (SCNEC): poorly differentiated, high grade, MR > 20 mitoses/2 mm^2^, Ki67 > 20% (often >70%), NEC, large cell type (LCNEC): poorly differentiated, high grade, MR > 20 mitoses/2 mm^2^, Ki67 > 20% (often >70%) and MiNEN: well or poorly differentiated, variable grade, variable MR, variable Ki67 [4].

Gastrointestinal tract cancers originating from the EGJ constitute a significant global health issue. Older age, male gender, long-standing gastroesophageal regurgitation disease (GERD), hiatal hernia size, and Barrett esophagus are strongly associated with higher grades of dysplasia and increased risk of esophageal adenocarcinoma development [8]. EGJ tumors are defined according to the Siewert classification as “tumors that have their center within 5 cm proximal and distal of the anatomical cardia” and are differentiated into three separate categories; type I is defined as adenocarcinoma of the distal esophagus with an epicenter of lesion 1–5 cm above gastro-esophageal junction, type II is defined as adenocarcinoma of the cardia with an epicenter of lesion up to 1 cm above or 2 cm below the gastro-esophageal junction, and type III is defined as sub-cardial adenocarcinoma with an epicenter of lesion 2–5 cm below the gastro-esophageal junction [9]. The eighth edition of the AJCC Cancer Staging Manual provided some modifications regarding tumors located at the EGJ. Specifically, tumors with an epicenter < 2 cm into the gastric cardia and involvement of the gastro-esophageal junction (type I and type II) are staged as esophageal adenocarcinoma, while tumors with an epicenter located > 2 cm into the gastric cardia and involvement of the gastro-esophageal junction (type III) are staged as gastric adenocarcinoma [10]. The Siewert classification along with the aforementioned recent modifications are crucial for determining whether an EGJ neoplasm will be handled as esophageal or gastric. Following the diagnosis of EGJ cancer, a necessary workup for clinical staging is required [11]. In case of metastasis, biopsy of metastatic disease should be performed as clinically indicated and could be used for biomarker testing [11]. Concomitantly, as the diagnosis of EGJ cancer is usually set at advanced stages, the implementation of more up-to-date precision imaging incorporating artificial intelligence algorithms could potentially be helpful in diagnosing EGJ cancer at earlier stages [12].

MiNENs originating from the esophago-gastric junction (EGJ) are extremely rare, and the exact frequency remains unknown. The gastric cardia mucosa contains various endocrine cells, which are mainly located on the surfaces of adjacent mucosal or parietal cells and could form the origin of the neuroendocrine malignant component of EGJ MiNENs. So far, only limited cases have been reported, none of which were diagnosed as having MiNEN prior to resection. MiNENs of the EGJ manifest with similar symptoms, endoscopic findings, and radiological findings compared to those of typical EGJ adenocarcinomas. Therefore, MiNENS are most often not diagnosed at the beginning but later on by histopathological and immunohistochemical examination of the resected specimen, if surgery is involved in the treatment algorithm. To date, available data regarding the treatment options of such tumors are limited, and there is no evidence on a proper treatment approach [1]. Prognosis is poor, with findings from a recent study including 401 patients suggesting that the 5-year disease-free survival is 51.1% [13]. In retrospective analyses, the grade and differentiation of the components directly impact the patients’ survival, and the neuroendocrine component has a key role in the prognosis [14,15].

In this manuscript, we discuss the clinical management of EGJ and gastric MiNENs based on a case of a 69-year-old female with an oligometastatic mixed neuroendocrine adenocarcinoma of the esophago-gastric junction (Siewert III), in which a multidisciplinary treatment approach resulted in a favorable outcome. The rationale behind the selection of this specific case is based not only on its rarity but also on the fact that there are currently no specific guidelines regarding the treatment of EGJ and gastric MiNENS. Further, we summarize the current literature on cases of EGJ MiNENs, which were treated as gastric as per the Siewert classification, focusing on demographics, tumor location, and data regarding patient survival. Since our case report concerns an oligometastatic MiNEN, we further analyze advanced EGJ-MiNENs under three categories: locally advanced, oligometastatic, and metastatic, by reporting on the therapeutic approaches that were followed along with details on patient survival. In this manuscript, we discuss the existing practices regarding the treatment of EGJ-MiNENs and propose an approach with a potential favorable outcome for cases where the MiNEN diagnosis is set early, during the pre-therapeutic phase.

## 2. Materials and Methods

### 2.1. Ethics Statement

This study was conducted in accordance with the Declaration of Helsinki. According to the Regulation of principles and operation of the research ethics committee of the National and Kapodistrian University of Athens, this study did not require the consent of the committee, as this case was not an experimental procedure. Regarding the case report, all necessary measures were taken to ensure patient integrity.

### 2.2. Case Presentation

**Figure 1:**
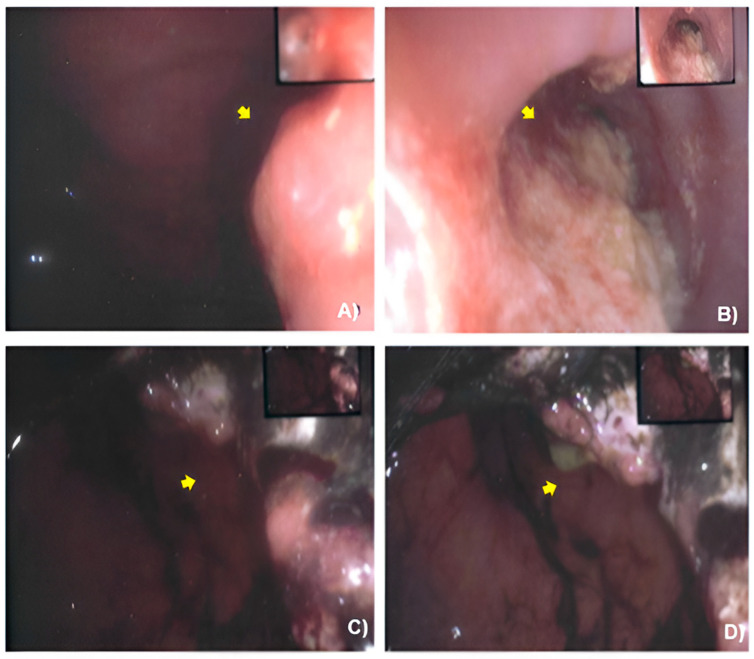
(**A**,**B**) Initial gastroscopy findings depicting an ulcerous lesion at the gastroesophageal junction and (**C**,**D**) the lesser curvature of gastric cardia (yellow arrows).

**Figure 2:**
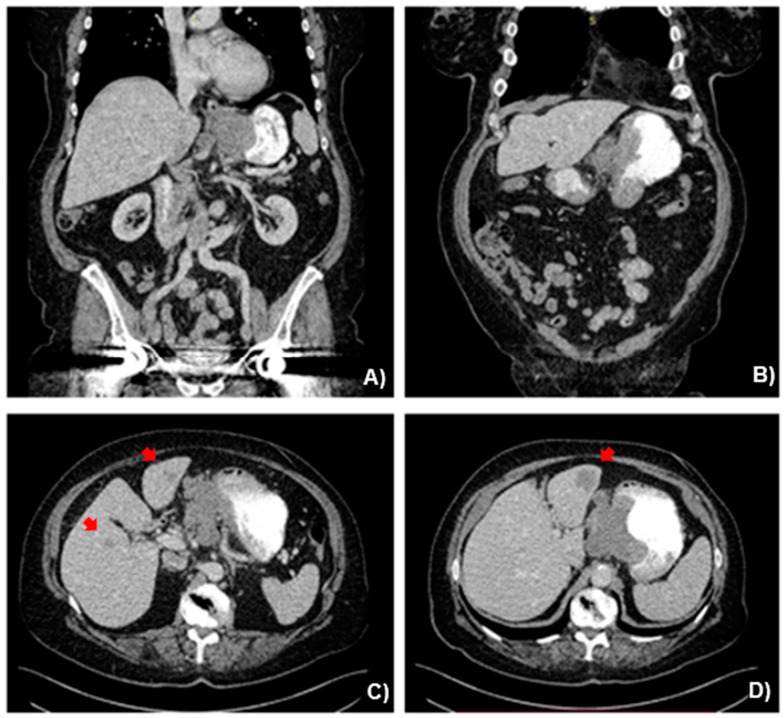
(**A**,**B**) Initial abdominal computed tomography in coronal and (**C**,**D**) axial views, depicting the gastric lesion and liver metastatic lesions (red arrows).

**Figure 3:**
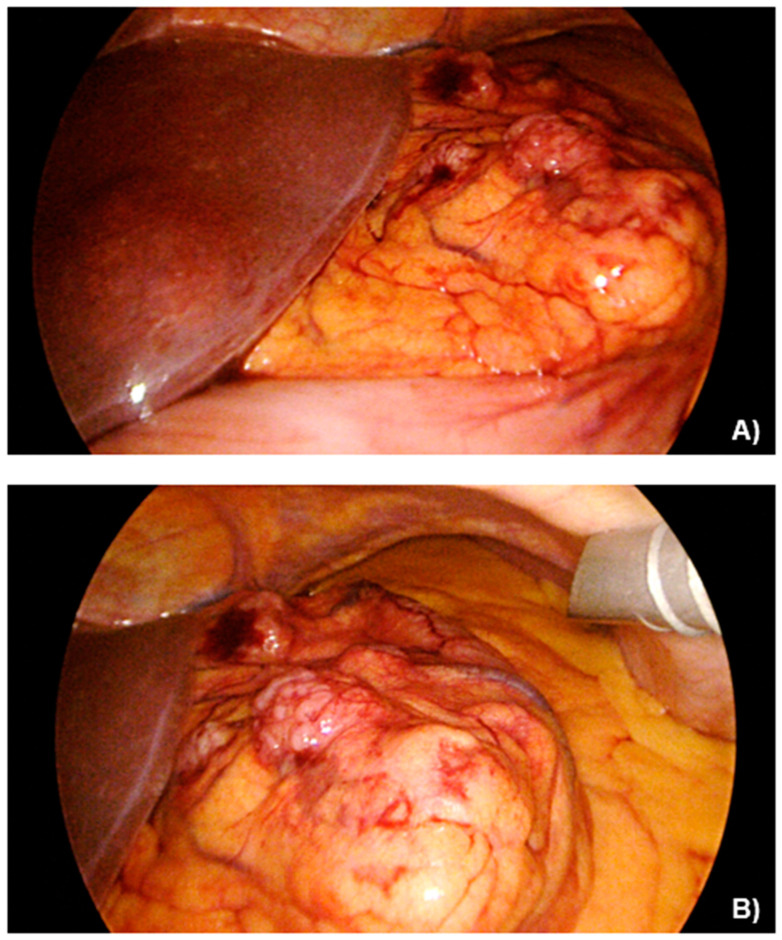
(**A**,**B**) Initial exploratory laparoscopy findings, negative for peritoneal seeding, with a visible liver metastatic lesion on the left hepatic lobe.

A 69-year-old female, with an unremarkable medical history, underwent an upper-GI endoscopy for progressive dysphagia for solid and liquids. A brittle ulcerous lesion with partial occlusion and signs of recent hemorrhage was detected at the EGJ with expansion to the lesser curvature of the gastric cardia, classified as a Siewert III tumor (Figure 1). The histopathological analysis of the endoscopic biopsies revealed a poorly differentiated invasive adenocarcinoma with HER-2 expression to be negative. The clinical staging included computed tomography (CT) (Figure 2) followed by a staging laparoscopy. Imaging demonstrated a locally advanced tumor (cT4) with a bulky locoregional lymph node packet (cN+) and three liver lesions consistent with hepatic metastases. In front of a possible oligometastatic state of disease, a staging laparoscopy took place to exclude peritoneal seeding and facilitate the application of a percutaneous feeding tube. During laparoscopy, macroscopic peritoneal seeding was excluded, while both the bulky lymph nodes at the lesser curvature and the largest liver metastasis at the left liver lobe could be visually detected (Figure 3). Remarkably, these tumorous lesions demonstrated a soft consistency in palpation with the laparoscopic forceps, which was not expected, as metastatic lesions from adenocarcinoma primary are anticipated to have a solid and hard texture. Therefore, a lymph node biopsy at the lesser curvature of the stomach was contacted in order to exclude different histopathology. The peritoneal cytology after collection of peritoneal washings proved to be negative.

Due to the worsening of dysphagia, the patient was immediately set under a systemic chemotherapy protocol of combined fluorouracil, leucovorin, and oxaliplatin (FOLFOX), based on the initial diagnosis of an oligometastatic Siewert III adenocarcinoma.

Remarkably, the histopathological analysis of the lymph node biopsy demonstrated the exclusive infiltration of a high-grade round-cell malignant neoplasm with neuroendocrine differentiation. Immunostaining was positive for NSE, vimentin, synaptophysin, and chromogranin A over 70%, all indicative of the presence of neuroendocrine cells. The Ki67 index was 95%. Pan-keratin markers were negative. Re-examination of the gastric biopsy with the application of immunostaining for neuroendocrine markers also revealed the presence of a neuroendocrine component, apart from the adenocarcinoma component, and at this point, the diagnosis of an oligometastatic mixed neuroendocrine adenocarcinoma of the esophago-gastric junction (Siewert III) was set.

### 2.3. Search of Literature 

Considering the low number of case reports of EGJ or gastric MiNENs and the lack of specific guidelines, a review of the current literature was conducted, in an attempt to gather the existent therapeutic approaches.

**Scheme 1:**
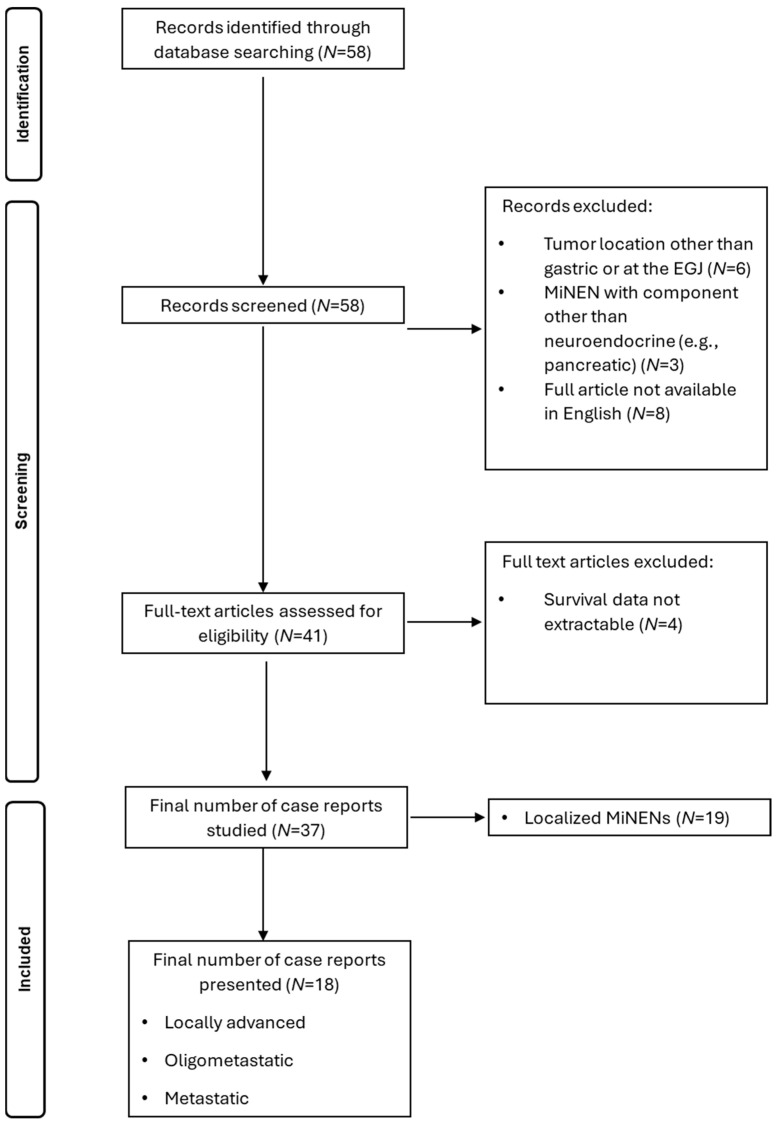
PRISMA flow diagram for selection of gastric and EGJ MiNENs. MiNEN = mixed neuroendocrine non-neuroendocrine neoplasm, *N* = number of studies, EGJ = esophagogastric junction, PRISMA = Preferred Reporting Items for Systematic Reviews and Meta-Analyses.

Thorough research was conducted on PubMed and Embase databases, using “Mixed Neuroendocrine Non-Neuroendocrine Neoplasm (MiNEN)”, “Mixed Adeno-neuroendocrine Carcinoma (MANEC)”, “Carcinoid tumor” and “Collision Tumor” as key words. A filter of case reports was implemented, and 58 articles were initially identified. Inclusion criteria involved case reports of MiNEN with gastric or EGJ location, which, according to the Siewert classification, were treated as gastric. Case reports not available in the English language with a tumor location other than gastric or EGJ and with histologic components other than adeno- and neuroendocrine were excluded, leading to 40 articles initially being assessed. Other exclusion criteria involved case reports with not enough data regarding patient survival. A total of 37 articles were finally assessed, reporting 39 patients in total. The PRISMA Algorithm for the selection of case reports is presented in Scheme 1. From 37 case reports, 19 referred to localized MiNENs and 18 to advanced MiNENs, the latter reporting 20 patients in total. Advanced MiNENs were separated and studied under three categories: locally advanced, oligometastatic, and metastatic. The 18 cases of advanced EGJ or gastric MiNENs ranged from 2012 to 2024 and were analyzed after a thorough review of the literature and implementation of exclusion criteria. Due to the rarity of such cases, we considered that exclusion of older cases could also potentially pose a selection bias, so we reported the tailored approaches that were followed in every case with respect to the publication date of each case report.

## 3. Results

### 3.1. Case Management

Regarding the management of our patient, the diagnosis of an oligometastatic mixed neuroendocrine adenocarcinoma of the esophago-gastric junction (Siewert III) was set, and the systemic chemotherapy was changed after one cycle of the FOLFOX protocol to a combination of etoposide with cisplatin, in order to tackle the more aggressive and metastatic neuroendocrine component. During the course of the systemic chemotherapy, the patient required a single hospitalization for right subclavian port thrombosis with an asymptomatic pulmonary embolism, and anticoagulation therapy was initiated. In the lack of established recommendations for MiNENs of the EGJ, as soon as the diagnosis of MiNEN of the EGJ was set and the neuroendocrine component was defined as the most aggressive one, as per the NCCN Clinical Practice Guidelines in Oncology, we first treated the neuroendocrine component with an induction chemotherapy regimen of etoposide and cisplatin [11].

**Figure 4:**
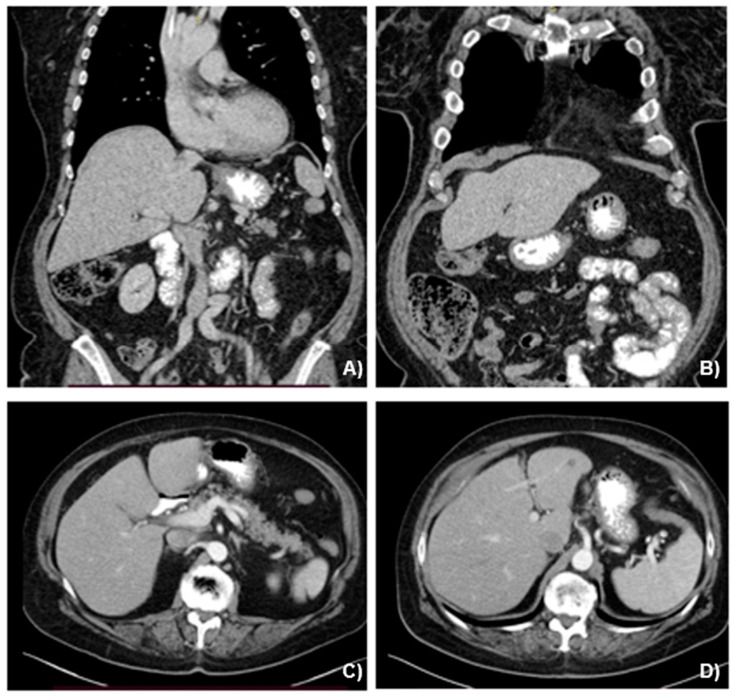
(**A**,**B**) Abdominal CT in coronal and (**C**,**D**) axial views after induction chemotherapy depicting significant tumor remission compared with the initial staging.

**Figure 5:**
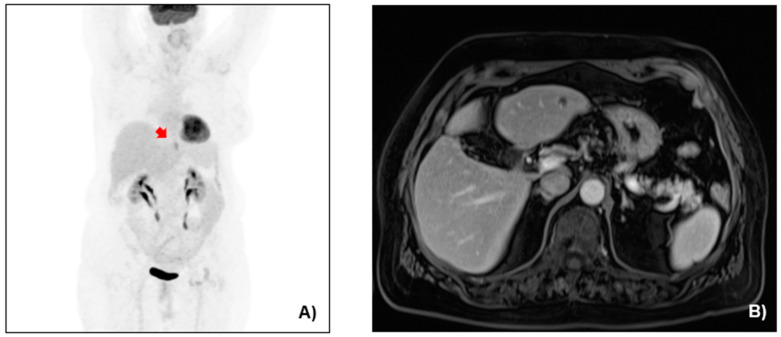
(**A**) FDG PET-CT in coronal view, with high SUV noticed at the GEJ (red arrow). (**B**) Abdominal MRI in axial view, showing a scar lesion at the site of the largest hepatic lesion at the left liver lobe.

**Figure 6:**
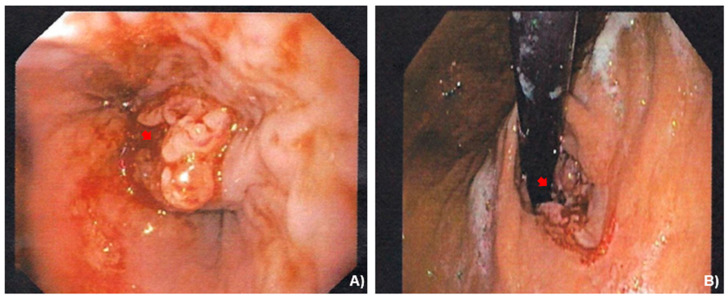
(**A**,**B**) Gastroscopy findings following completion of induction chemotherapy depicting a significant tumor remission with a single ulcer at the gastric cardia (red arrows).

**Figure 7:**
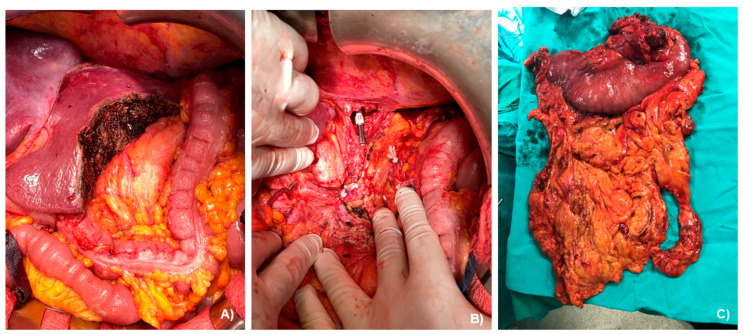
(**A**) Surgical situs post-gastrectomy and atypical left hepatectomy. (**B**) Impression of the celiac trunk post-resection showing the D2-lympadenectomy and ligation of left gastric artery. (**C**) Impression of the en bloc resected surgical specimen (extended total gastrectomy with D2 lymph node dissection).

After the completion of a total of six cycles of chemotherapy, the re-staging CT showed major remission of the primary tumor and the metastatic sites (Figure 4). The imaging was complemented with FDG PET-CT and MRI-Liver (Figure 5). High SUV was noticed solely at the EGJ on the FDG PET-CT (Figure 5A). Out of the known hepatic lesions initially present, two were not visible on MRI and one had significantly reduced in size (from 2.6 to 0.7 cm) (Figure 5B). There was also major remission of the patient’s bulky lymph node packet at the lesser curvature. Gastroscopy findings following the completion of induction chemotherapy depicted a significant tumor remission with a single ulcer at the gastric cardia (Figure 6). Given the significant tumor response after induction chemotherapy, with the sole presence of residual active tumor exclusively at the EGJ together with the good performance status of the patient, a conversion surgical resection was recommended. Following MDT discussion, the patient was finally considered eligible for radical surgical resection, in which we decided to include surgical local management of all previous liver metastatic sites. The patient underwent an extended total gastrectomy with D2 lymph node dissection together with metastasectomy of the remaining liver metastatic lesions through an atypical left hepatectomy along with radiofrequency ablation (RFA) of a single scar tissue on the right liver, which was spotted at the site of previous hepatic metastasis and was evaluated using intraoperative liver ultrasound. Intraoperative images along with the image of the resected surgical specimen are shown in Figure 7.

**Figure 8:**
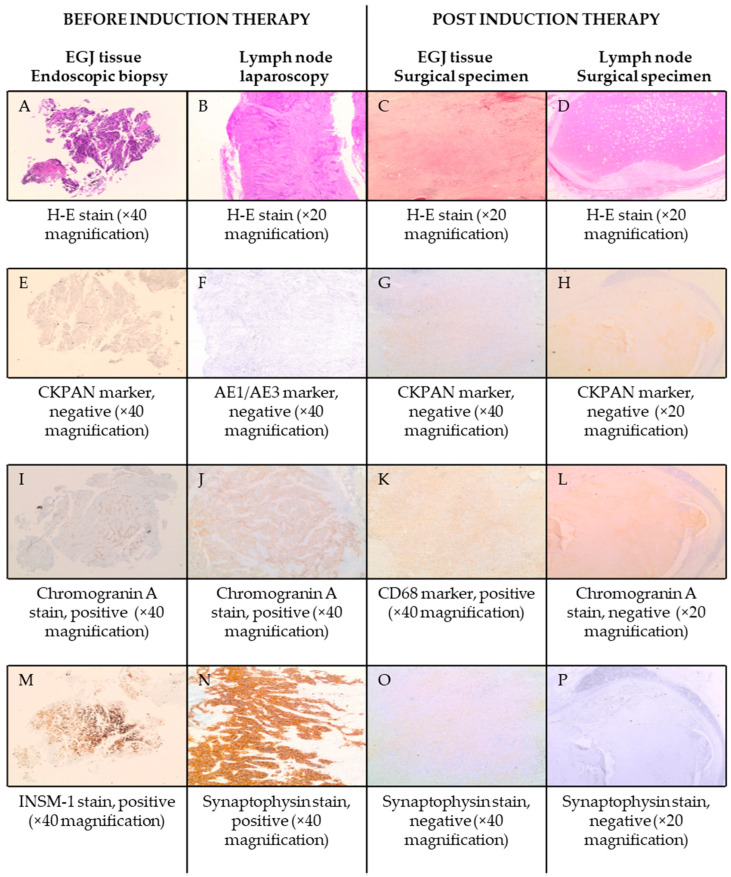
Histologic images from primary EGJ tumor and lymph node, before and after induction chemotherapy. Hematoxylin–eosin stains of high-grade neuroendocrine carcinoma on biopsy of stomach cardia and lymph node before induction therapy (images (**A**,**B**), magnification ×40 and ×20, respectively), with immunohistochemical negativity for CKPAN or AE1/AE3 (images (**E**,**F**), magnification ×40, respectively) and positivity for Chromogranin A (Images (**I**,**J**), magnification ×40), INSM-1 or Synaptophysin (images (**M**,**N**), magnification ×40). Histologic images of stromal (images (**C**,**G**,**O**), magnification ×40) and lymph node (images (**D**,**H**,**L**,**P**), magnification ×20) regression with absence of CKPAN, Chromogranin A, and Synaptophysin staining. There is diffuse histiocytic stromal infiltration, as becomes apparent with CD68 expression (image (**K**)), magnification ×40).

**Figure 9:**
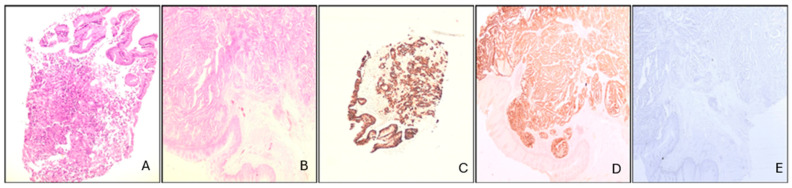
Histologic images from adenocarcinoma before and after induction of therapy. Hematoxylin–eosin stain of well-differentiated adenocarcinoma on biopsy of stomach cardia before induction therapy (image (**A**), magnification ×100, cropped image), with immunohistochemical positivity on CKPAN (image (**C**), magnification ×100, cropped image). Hematoxylin–eosin stain of well-differentiated adenocarcinoma on surgical specimen of esophagogastrectomy (image (**B**), magnification ×20) with immunohistochemical positivity in CDX2 (image (**D**), magnification ×20) and negativity in Chromogranin A (image (**E**), magnification ×20).

The histopathological analysis of the resected surgical specimen showed complete pathological remission of the neuroendocrine component and detected remnant of the adenocarcinoma tumor cells at the site of the primary tumor at the gastric cardia at stages ypT2, N0 (0/41), M0, pN0, pV0, pL0, and R0. HER 2 immunochemistry was negative. Histopathological images from pre- and post-treatment specimens are presented in Figure 8 and Figure 9. The complete pathological regression of the neuroendocrine component in lymph node tissue is depicted in Supplementary Figure S1 (before induction therapy) and Figure S2 (after induction therapy). The postoperative course was uneventful.

Following a recovery period of four weeks postoperatively, an adjuvant systemic treatment based on molecular tumor analysis was applied. Due to the exclusive presence of residual adenocarcinoma of the primary lesion and a positive PD-L1-/CPS-Score of 6, we administered systemic chemotherapy with FOLFOX accompanied by immunotherapy with Nivolumab for three months, followed by maintenance therapy with Nivolumab monotherapy. Apart from grade II thyroiditis, which was managed successfully by an endocrinologist, no other serious adverse effects occurred.

**Figure 10:**
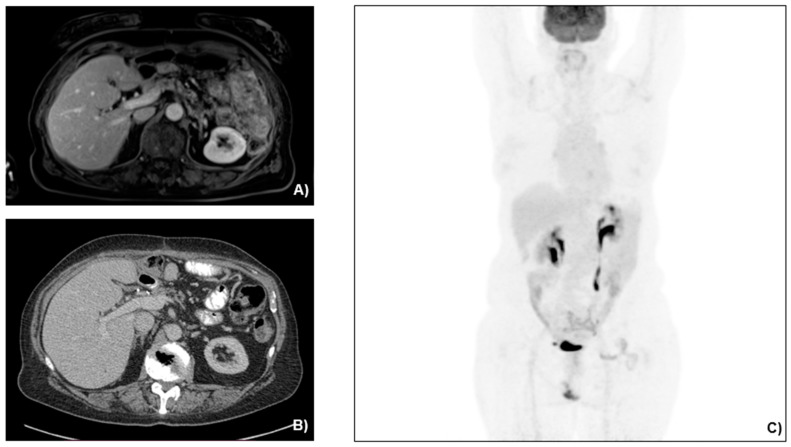
(**A**) Abdominal MRI, (**B**) abdominal CT, (**C**) FDG PET-CT at 3-years follow-up post-surgery with absence of recurrent disease.

The patient is disease-free at the 3-year follow-up, as shown in Figure 10. She remains in good condition and with a stable weight under nutritional supervision, signifying an overall successful multidisciplinary treatment. An alternative option to our management would be the continuation of chemotherapy in a palliation setting despite the major response. This alternative, however, would contain risks associated with possible chemo-resistance and future tumor progression along with a suboptimal therapeutic regimen for the second component, the adenocarcinoma. There was a concern regarding the recurrence of the neuroendocrine component immediately post-operatively [16], which we addressed with a strict observation protocol.

### 3.2. Literature Review

Out of 39 patients, 28 (71.8%) were male and 11 (28.2%) were female. The mean age of diagnosis was 67.6 years. Regarding tumor anatomical location, 33 patients were presented with gastric MiNEN (84.6%), and 6 with MiNEN at the EGJ (15.4%). Regarding mortality rates, out of 39 patients in total, 12 have passed away (30.8%), 17 are on an ongoing surveillance protocol (43.6%), and 10 have exceeded 2-year survival (25.6%).

From 37 case reports, 19 referred to localized MiNENs and 18 to advanced MiNENs, the latter reporting 20 patients in total. Advanced MiNENs were separated and studied under three categories: locally advanced (*N* = 13), oligometastatic (*N* = 3), and metastatic (*N* = 4). Data are presented in Table 1.

From the thirteen patients with locally advanced gastric MiNEN [17,18,19,20,21,22,23,24,25,26,27,28], only one received neoadjuvant chemotherapy with etoposide plus carboplatin [19]. They all underwent total gastrectomy, followed by adjuvant regimens that targeted the most aggressive component. Specifically, mFOLFOX (oxaliplatin, 5-fluorouracil, and leucovorin) was mostly used to treat the adenocarcinoma component, whereas platinum-based therapy primarily treated the neuroendocrine component. Interestingly, anti-PD1 treatment was successfully administered in two patients will locally advanced disease unresponsive to standard chemotherapy that progressed to metastasis [17,19].

Two patients [18,29] fulfilled the criteria of oligometastatic disease (OMEC), defined as 1 organ with ≤3 metastases or 1 involved extra-regional lymph node station. An organ-specific definition of oligometastatic disease includes ≤3 unilobar liver metastases, ≤3 unilateral lung metastases, unilateral adrenal gland involvement, or 1 bone or soft tissue metastasis [30]. They presented with hepatic metastases. In all of them, the neuroendocrine component was identified at the site of metastasis. Woo et al. presented a case of an oligometastatic gastric MiNEN treated successfully with irinotecan plus cisplatin (IP) as induction chemotherapy, total gastrectomy plus left hepatectomy, and adjuvant IP therapy. The patient was disease-free at 3 years after diagnosis [18]. Regarding the second case, the patient had tumor infiltration of regional lymph nodes and one liver metastasis at the left liver lobe and could potentially be a candidate for cytotoxic chemotherapy [29]. However, the patient underwent gastrectomy and refused any chemotherapy regimen. Overall survival did not exceed 2.5 months.

Five case reports presented patients with metastatic disease, with multiple liver metastases and distant lymph node metastases [31,32,33,34,35]. Two patients received an EP regimen without response, one patient received a scheme of S-1, CDDP and Trastuzumab, and two were offered palliative treatment due to multiple comorbidities. Due to disease progression, overall survival ranged between 3 and 9 months.

Lopez et al. summarizes the current treatment options regarding MiNENS in the gastroenteropancreatic tract [1]. Interestingly, it is highlighted that the percentage of each component, with emphasis on the 30% threshold, should be determined in each case. However, even a minor (i.e., <30%) poorly differentiated neuroendocrine carcinoma (PDNEC) component could impair prognosis, indicating that the current definition could undergo changes in the future [1,18]. Overall, it appears that current practices are in line with our suggestions. Regarding oligometastatic disease, platinum-based chemotherapy combined with etoposide or irinotecan is preferred as the induction regimen, followed by gastrectomy and left hepatectomy. Performance status is crucial in deciding whether patients are eligible to commence chemotherapy. This can only strengthen the need for further research on the field of oligometastatic MiNENs, in order to standardize a therapeutic protocol with the most favorable outcome for the patients.

**Table 1:** Data from case reports of locally advanced, oligometastatic, and metastatic gastric or EGJ mixed neuroendocrine adenocarcinomas.

Authors	Locally Advanced	Oligo-Metastatic	Metastatic	Neuroendocrine to Non-Neuroendocrine Ratio	Induction Chemotherapy Regimen	Surgery	Adjuvant Therapy	Other Therapies	Overall Survival
Zheng et al., 2024 [17]	Yes	No	No	50%/50%	No	Total gastrectomy pT3N1M0	Delayed due to postoperative condition	Progression with liver metastasis treated with etoposide, cisplatin, toripalumab (6 cycles)	Ongoing at 1 y
Lun Tao Woo et al., 2022 Patient 1 [18]	Yes	Left liver lobe	No	80%/20%	Irinotecan plus cisplatin (4 cycles)	Total gastrectomy and partial left liver resection pT1aN1M0	Irinotecan plus cisplatin (2 cycles)	No	Ongoing at 3 y
Lun Tao Woo et al., 2022 Patient 2 [18]	Yes	No	No	80%/20%	No	Total gastrectomy pT4aN3aM0	Irinotecan plus cisplatin (6 cycles)	VATS due to recurrence at 2 y with metastatic lesions at the lower lobe of the right lung	Ongoing at 7 y
Deliwala et al., 2021 [32]	Yes	No	Left liver lobe, paraaortic lymph nodes	N/A	Carboplatin plus etoposide (2 cycles)	No	No	Capecitabine plus temozolomide following osseous metastases	3 m, disease progression
Millet et al., 2021 [33]	Yes	No	Liver, retroperitoneal adenopathy, mesenteric implants	N/A	No	No	No	Palliative treatment	Unknown, disease progression
Ricco et al., 2020 [19]	Yes	No	No	60%/40%	Carboplatin plus etoposide (3 cycles)	Total gastrectomy pT4N1M0	mFOLFOX6 (6 cycles)	Multiple chemotherapy regimens, radiation therapy and antiPD-1 treatment due to disease progression	Ongoing
Lin et al., 2019 [20]	Yes	No	No	N/A	No	Total gastrectomy	Oxaliplatin plus capecitabine (4 cycles)	Radiofrequency Ablation due to hepatic metastasis at 4 cycles, paclitaxel plus Cisplatin (6 cycles), Abatinib	Ongoing
Golombek et al., 2019 [34]	Yes	No	Liver, distant lymph nodes	N/A	Etoposide plus cisplatin	No	No	No	9 months, disease progression
Tang et al., 2017 Patient 1 [21]	Yes	No	No	70%/30%	No	Total gastrectomy pT4aN1M0	Etoposide plus cisplatin	No	Ongoing at 6-month follow up
Tang et al., 2017 Patient 2 [21]	Yes	No	No	30%/70%	No	Total gastrectomy pT4aN1M0	No treatment due to general weakness	No	6 m, general weakness
Kheiri et al., 2017 [22]	Yes	No	No	N/A	No	Partial gastrectomy T4aN2M0	mFOLFOX (12 cycles)	No	Ongoing at 2 y follow up
Pham et al., 2017 [35]	Yes	No	Liver, pancreas, distant lymph nodes	80–90%/10–20%	No	No	S-1, CDDP, Trastuzumab (5 cycles)	Yes	7 m, disease progression
Ambesh et al., 2017 [23]	Yes	No	No	60%/40%	No	Ivor-Lewis Esophagogastrectomy pT3N1M0	No	No	Unknown
Cazzo et al., 2016 [24]	Yes	No	No	40%/60%	No	Total gastrectomy pT4N0M0	5-fluorouracil plus leucovorin (5 cycles) plus radiation therapy	No	Ongoing at 1 y follow up
Taguchi et al., 2015 [25]	Yes	No	No	30%/70%	No	Total gastrectomy	Tegafur plus uracil	No	Ongoing at 1 y and 4 m
Gurzu et al., 2015 [26]	Yes	No	No	40%/60%	No	Total gastrectomy pT4N3b	Patient refused treatment	No	5 m, disease progression
Levi et al., 2014 [27]	Yes	No	No	30%/70%	No	Total gastrectomy pT4aN0M0	Cisplatin, doxorubicin, and vincristine	No	Ongoing at 6 m follow up
Zhang et al., 2014 [29]	Yes	Left liver lobe	No	N/A	No	Total gastrectomy and left liver resection	Patient refused treatment	No	2.5 m, disease progression
Pericleous et al., 2012 [31]	Yes	No	>3 liver lesions	N/A	No	No	No	Somatostatin analogues	Unknown
Miguchi et al., 2012 [28]	Yes	No	No	N/A	No	Total gastrectomy	S-1	No	Ongoing at 6 m follow up

## 4. Discussion

Mixed neuroendocrine adenocarcinomas of the esophago-gastric junction or stomach present rare and challenging cases. The challenging points include, on the one hand, their rather late diagnosis, mostly after surgical resection in the final histopathology, if surgery is involved, and, on the other hand, their advanced stage with usually metastatic disease, which excludes approaches with a curative intention. Limited data exist, however, on the oncological management of EGJ-MiNENs, when diagnosis is set early or in a locally advanced or oligometastatic state. Despite MiNENs being recognized as a separate clinical entity by the WHO and the European Neuroendocrine Tumor Society, no specific validated treatment guidelines are currently available [1]. Regarding the first-line chemotherapy for advanced MiNENs, there is currently no agreement [9]. Because the clinical behavior of gastric NECs resembles that of small-cell lung cancer, which is responsive to etoposide and cisplatin (EP), EP is used as a combination therapy in patients with extra-pulmonary NEC including gastric MiNEN [36]. The TOPIC-NEC Phase 3 Randomized Clinical Trial evaluated the effectiveness of Etoposide and Cisplatin (EP) vs. Irinotecan and Cisplatin (IP) as therapy for patients with advanced neuroendocrine carcinoma of the digestive system, concluding that both EP and IP remain the standard first-line chemotherapy options [37].

Our clinical case provides insight into significant issues concerning clinical management of EGJ MiNENs that could be taken into consideration. The staging laparoscopy along with the surgeon’s experience and threshold to perform a lymph node biopsy resulted in the detection and diagnosis of the mixed nature of the tumor with a neuroendocrine component, which appeared to be the metastasized component. Considering that MiNENS are usually diagnosed in surgical specimens after major resections late during the oncological course, the current histological detection of the neuroendocrine component during the early steps of the oncological management changed the oncological course and determined the positive oncological outcome in our case. The neuroendocrine component seems to often be missed out when neuroendocrine markers are not routinely used during examination of the biopsy specimen and not because of the potentially small proportions of the biopsy specimen [38]. In order to address this issue and minimize the risk of misdiagnosis, we suggest that the histopathological assessment of the endoscopic biopsies of EGJ lesions should include at least one neuroendocrine marker, especially if the solid component of tumor cells is discovered at hematoxylin and eosin staining. In our case, even from the small biopsy specimen that was taken, the presence of neuroendocrine cells was verified when specific staining was used, thus playing a key role in the induction chemotherapy regimen of etoposide and cisplatin that our patient received. This course of events also helped us realize and abide by a more complete approach, in order to routinely use a neuroendocrine marker in future similar cases. Further, the decision to substitute the initial induction chemotherapy in order to attack the more aggressive and metastatic neuroendocrine component was crucial, since the major response of the tumor lesions to the adjusted chemotherapy enabled conversion surgery. Additionally, the recognition of an oligometastatic state during initial staging enabled an oncological approach with curative intention, which facilitated the decision for radical conversion surgery, including metastasectomy, due to the tumor regression.

In a retrospective critical review of the case presented, the lessons learnt could be summarized as follows: (a) the histopathological assessment of the endoscopic biopsies of EGJ lesions should include at least one neuroendocrine marker, especially if the solid component of tumor cells is discovered following hematoxylin and eosin staining. This also aligns with the findings of Jepsen et al. [38] regarding immuno-histological staining with neuroendocrine markers. (b) Staging laparoscopy should be considered during initial staging for locally advanced tumors, even in cases with an oligometastatic state, based on the latest European clinical practice guidelines for the definition, diagnosis, and treatment of oligometastatic esophagogastric cancer (OMEC-4) [30]. (c) Oncological decisions on which the induction chemotherapy regimen is decided should take into consideration the percentage of the neuroendocrine component at the primary lesion along with further histopathological features such as the proliferation index. (d) Conversion surgery with curative intention after a major tumor response following induction chemotherapy should not be excluded, even for initially oligo-metastatic disease states. Regarding surgical resection, both the primary tumor and the metastatic lesions should be addressed in patients with initially oligometastatic disease. Decisions should be made during MDT discussion. (e) To ensure a holistic approach to such cases, patients could also benefit from molecular analysis to detect target therapies and prognostic factors. Examination should at least include, apart from the HER2 expression, the status of microsatellite instability MSS and the expression of PDL1 in tumor tissue along with calculation of the combined positive score (CPS) and tumor proportion score (TPS), in order to facilitate the addition of immunotherapy agents. Although a single case report could under no circumstances fulfil the criteria to be solely followed in decision-making, we hope that our case will help raise interest for EGJ or gastric MiNENs and inspire more robust and expanded case series that will reach more concrete conclusions.

The literature review on similar cases of EGJ or gastric MiNENs revealed a limited number of reported cases. Out of the 39 patients who were diagnosed with EGJ or gastric MiNEN, 20 of them (51.3%) suffered from advanced disease, which could be either locally advanced, oligometastatic, or metastatic. Notably, due to non-specific symptomatology such as common EGJ adenocarcinomas, a significant number of patients seem to seek medical advice when dysphagia is present and the tumor is already at a more advanced disease stage. The neuroendocrine component does not provide specific symptomatology to facilitate earlier detection. Following the treatment strategies that were implemented in the reported cases, it can be supported that once the diagnosis of a MiNEN is set, the management is modified to attack the most aggressive tumor component, which, in most cases, is the neuroendocrine tumor. In all cases, a multidisciplinary approach was found to be applied, involving the management of different specialties such as surgical oncologists, oncologists, and pathologists for the patient’s best interest in a tailored approach. In cases such as that explored by Woo et al., where there was significant tumor remission following induction chemotherapy, consideration of eligibility criteria for a surgical operation proved to play a valuable role in extending patient survival. Thus, it is crucial to consider conversion surgery with curative intent in such cases. That should always be with respect to the patient’s overall performance status and personal wishes and follow MDT discussion. The low number of cases that are included in this review can be interpreted in two ways: one the one hand, MiNENs indeed represent rare neoplasms, an issue that acts as an inhibitory factor for conducting studies with larger samples in order to reach a diagnosis and therapy consensus. On the other hand, our review of the literature shows that physicians share general principles regarding the therapeutic approach of MiNEN, such as targeting the aggressive neuroendocrine component rather than the adenocarcinoma, or having a low threshold to proceed with conversion surgical resections when a major response to induction systemic therapy is administrated even in an oligometastatic disease state. These general principles could form the basis for current evidenced-based approach regarding such cases.

## 5. Conclusions

In conclusion, MiNENs of the EGJ or stomach are rare and aggressive neoplasms with heterogenous histological and morphological characteristics. The key to successful management is the early detection of the mixed nature of the malignant tumor. Therefore, immunostaining of at least one neuroendocrine marker on endoscopic biopsies in EGJ tumors should facilitate early diagnosis. Both the neuroendocrine and non-neuroendocrine components of the tumor should be taken into consideration, but according to the current literature, the therapeutic approach should be based on the high-grade neuroendocrine component as the treatment target [1]. Laparoscopy with synchronous biopsy of suspicious lesions at the pre-therapeutic phase could prove valuable in decision making. A multidisciplinary approach should aim for a personalized therapeutic plan based on patients’ characteristics and tumor response. Conversion surgery, including metastasectomy in cases of oligometastatic disease, may provide an option to increase patient survival. Due to the rarity of MiNENs of the esophagogastric junction and given the fact that there are currently no specific guidelines for specific treatment, we consider that this case of successful multimodal management provides a useful example for other practitioners and enhances the limited existing literature. The presentation of case series and meta-analyses remains critical for standardizing a therapeutic protocol for this tumor entity.

## Data Availability

The raw data leading to the results of this article are available on request from the corresponding author.

## Supplementary Materials

The following supporting information can be downloaded at https://www.mdpi.com/article/10.3390/jcm14051503/s1: Figure S1: Histologic Images of neuroendocrine carcinoma on lesser curvature lymph node biopsy before induction chemotherapy; (A,B): Hematoxylin-eosin stain (magnification ×20 and ×200 respectively), (C,D): Immunohistochemistry positive for Synaptophysin (magnification ×40) and Ki67 (magnification ×100) respectively; Figure S2: Histologic features of regression of the neuroendocrine carcinoma in lymph node surgical specimen after induction chemotherapy; Hematoxylin-eosin stain of lymph nodes from surgical specimen (Images A, B, C-magnification ×20), Synaptophysin and Chromogranin negativity (Images D and E respectively-magnification ×20).

## Abbreviations

The following abbreviations are used in this manuscript:

MiNEN	Mixed neuroendocrine–non-neuroendocrine neoplasm
EGJ	Esophagogastric junction
MDT	Multidisciplinary team
MANEC	Mixed adenoneuroendocrine carcinoma
