# Supplementary material for: Oligometastatic Mixed Neuroendocrine Adenocarcinoma of the Esophago-Gastric Junction: A Case of Successful Multidisciplinary Management, the Lessons Learnt and Review of the Literature
Source: J Clin Med. 2025 Feb 24;14(5):1503. doi: 10.3390/jcm14051503 (PMC11899748; doi:10.3390/jcm14051503)
Supplement: Supplementary file 1 [file jcm-14-01503-s001.zip › jcm-3484019-supplementary.pptx]

## Slide 1
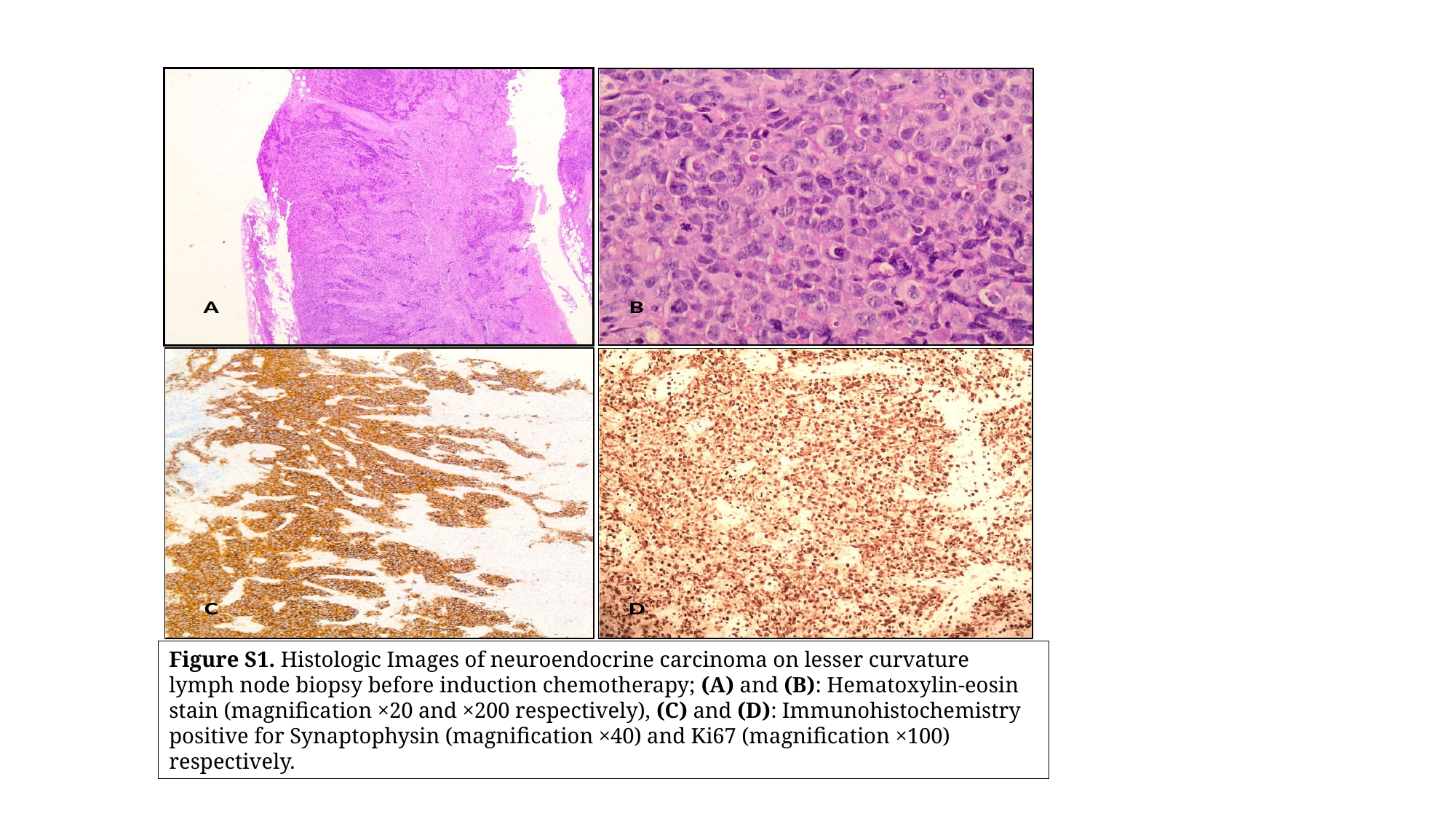

Figure S1. Histologic Images of neuroendocrine carcinoma on lesser curvature lymph node biopsy before induction chemotherapy; (A) and (B): Hematoxylin-eosin stain (magnification ×20 and ×200 respectively), (C) and (D): Immunohistochemistry positive for Synaptophysin (magnification ×40) and Ki67 (magnification ×100) respectively.

## Slide 2
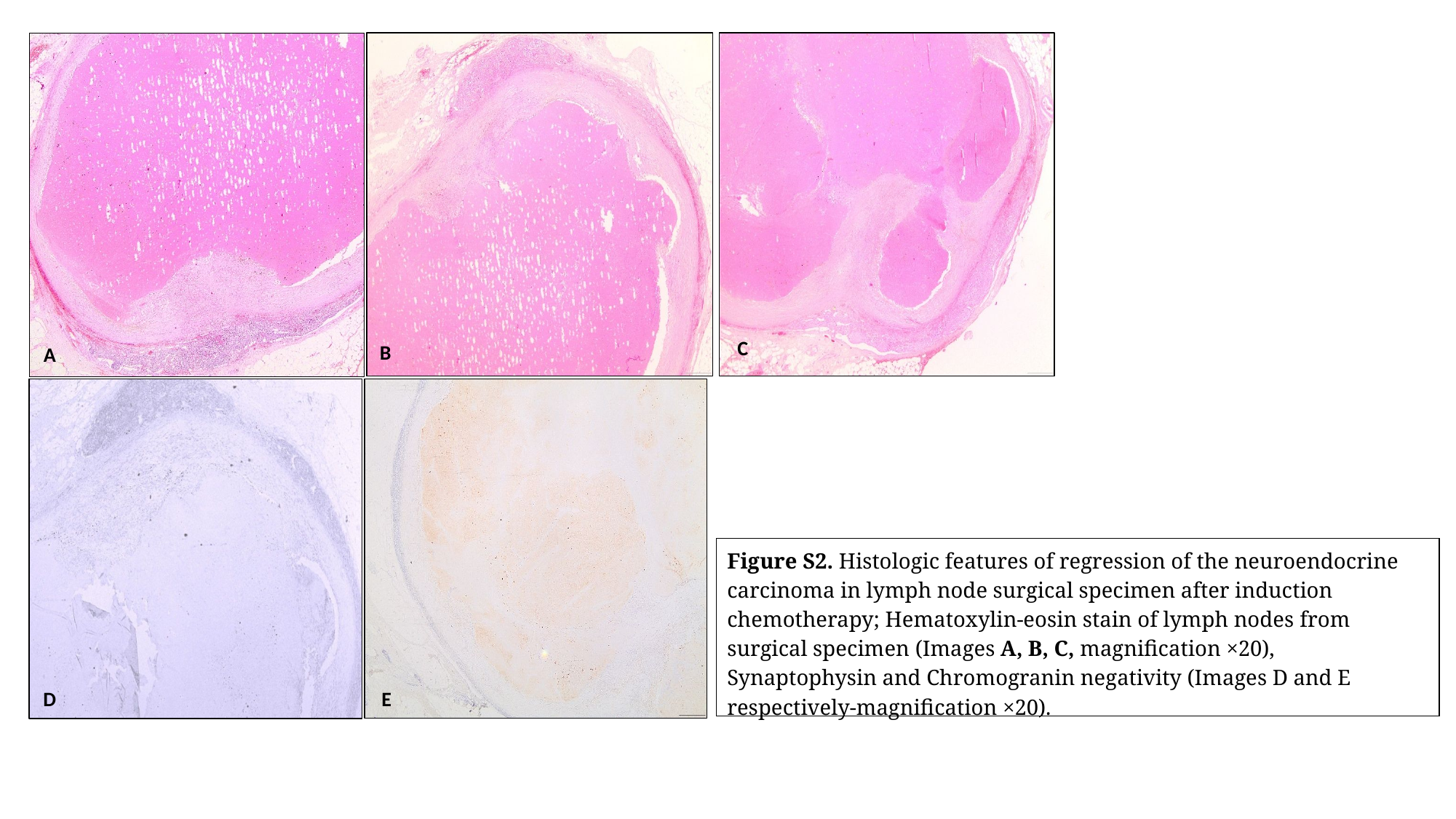

Figure S2. Histologic features of regression of the neuroendocrine carcinoma in lymph node surgical specimen after induction chemotherapy; Hematoxylin-eosin stain of lymph nodes from surgical specimen (Images A, B, C, magnification ×20), Synaptophysin and Chromogranin negativity (Images D and E respectively-magnification ×20).
